# Scientometric Research and Critical Analysis of Gait and Balance in Older Adults

**DOI:** 10.3390/s24103199

**Published:** 2024-05-17

**Authors:** Qian Mao, Wei Zheng, Menghan Shi, Fan Yang

**Affiliations:** 1 School of Design, The Hong Kong Polytechnic University, Hong Kong; 2 Department of Computer Science and Technology, Tsinghua University, Beijing 100190, China; 3 Lancaster Imagination Lab, Lancashire, Lancaster LA1 4YD, UK; 4 Electrical and Electronic Engineering Department, The Hong Kong Polytechnic University, Hong Kong

**Keywords:** gait and balance, global ageing, scientometric method, interrelated literature research, clustering analysis

## Abstract

Gait and balance have emerged as a critical area of research in health technology. Gait and balance studies have been affected by the researchers’ slow follow-up of research advances due to the absence of visual inspection of the study literature across decades. This study uses advanced search methods to analyse the literature on gait and balance in older adults from 1993 to 2022 in the Web of Science (WoS) database to gain a better understanding of the current status and trends in the field for the first time. The study analysed 4484 academic publications including journal articles and conference proceedings on gait and balance in older adults. Bibliometric analysis methods were applied to examine the publication year, number of publications, discipline distribution, journal distribution, research institutions, application fields, test methods, analysis theories, and influencing factors in the field of gait and balance. The results indicate that the publication of relevant research documents has been steadily increasing from 1993 to 2022. The United States (US) exhibits the highest number of publications with 1742 articles. The keyword “elderly person” exhibits a strong citation burst strength of 18.04, indicating a significant focus on research related to the health of older adults. With a burst factor of 20.46, Harvard University has made impressive strides in the subject. The University of Pittsburgh displayed high research skills in the area of gait and balance with a burst factor of 7.7 and a publication count of 103. The research on gait and balance mainly focuses on physical performance evaluation approaches, and the primary study methods include experimental investigations, computational modelling, and observational studies. The field of gait and balance research is increasingly intertwined with computer science and artificial intelligence (AI), paving the way for intelligent monitoring of gait and balance in the elderly. Moving forward, the future of gait and balance research is anticipated to highlight the importance of multidisciplinary collaboration, intelligence-driven approaches, and advanced visualization techniques.

## 1. Introduction

With the intensification of ageing in many countries around the world, by 2050, the number of elderly people will reach 1.5 billion, accounting for 16% of the world’s total population [1]. Elderly health has progressively emerged as a crucial topic of study. The goal of research has been to find better techniques to keep track of older patients’ health and lower their risk of illness and falls. Balance and gait have been intensively investigated because they are considered key factors in predicting health status and fall risk [2,3,4,5,6].

Human gait is an essential component of daily life and is a significant predictor of general health [7,8,9]. People’s ability to walk and gait speed may gradually deteriorate as they get older [10,11,12]. Hence, the study of gait, which includes the posture and behavioural traits of the human body while walking, has grown in significance, especially in relation to the health and wellbeing of older persons [12,13,14]. Gait is now a factor in determining how well an aged person is doing [15,16]. Balance was described as a sophisticated bodily function used to carry out tasks requiring equilibrium [17,18]. While falls are the primary cause of morbidity in older adults, given their longer life expectancies and more active lifestyles, it is important to spot any changes in their gait patterns in order to decrease the frequency of falls as well as to make it simpler for older adults to access diagnostic tools for reliable fall predictors, and finally, to develop a strategy to prevent such falls [16,19,20,21].

The global aging population has raised concerns about gait and balance, underscoring their vital role in preserving seniors’ health [22,23,24,25]. Many different parameters have been used to characterise and examine gait and balance, such as energy metabolism, kinematics, kinetics, and gait cycle [26,27,28]. Approximately one-fifth of individuals aged 65 and above rely on walkers for mobility assistance, while around 30% encounter challenges with activities like climbing stairs or walking longer distances. The prevalence of gait impairments tends to rise with age, evident in both acute hospital environments and long-term care facilities [1,29]. Research indicates that over a quarter of individuals aged 70 to 74 exhibit abnormal gait patterns, with the percentage increasing to nearly 60 for those aged 80 to 84 [19]. It is crucial to comprehend the stability of gait and the distribution of gravity in order to successfully avoid falls using sensors and other devices because gait and balance deficiencies are thought to be the primary risk factors for falls in older persons [30,31,32]. Few articles, however, provide a thorough appraisal of the state of gait and balance research from 1993 through 2022. 

Gait and balance are frequently investigated to assess the recovery of patients who have suffered from trauma or disease. For instance, the evaluation of gait analysis, balance, and vestibular testing can reveal subtle changes in gait and balance in patients with traumatic brain injury (TBI), facilitating the exploration of the interplay between gait and balance deficits after TBI [26]. Functional gait disorders are prevalent among patients referred to Movement Disorders Clinics, with studies indicating that 2–20% of these patients exhibit psychogenic movement disorders. Interestingly, approximately 40% of individuals diagnosed with psychogenic movement disorders also present with gait abnormalities. This underscores the complexity of gait disorders and highlights the need for comprehensive assessment and management strategies in clinical settings.

The scientific method is still required to examine the research development and the state of gait and balance in older individuals by counting all the literature and analysing the characteristics [33]. In this study, scientific statistics on the literature in the field of gait and balance are used for the first time. The development summary and trend prediction of gait and balance are then realised using the relevant information, such as literature keywords and subject distribution. The current paper offers a more thorough and objective evaluation of the most recent studies on gait and balance in older persons than earlier reviews have as well as reliable forecasts. This study aims to comprehensively explore and analyse the application areas of gait and balance of older adults through scientometric research and critical analysis, providing detailed descriptions and insights into these crucial aspects of human locomotion.

The following sections make up the review: The study’s methodology is described in Section 2 of this article. As established by a CiteSpace analysis, Section 3 analyses the significant publications and conferences on the subject of gait and balance from WoS as well as the major source nations and organisations, core authors, and keywords. In Section 4, an analysis of the experimental techniques utilised in gait and balance studies from 1993 to 2022 is presented. Gait and balance research participants are examined in Section 5. The final section provides a summary of the review.

## 2. Research Methodology

**Figure 1:**
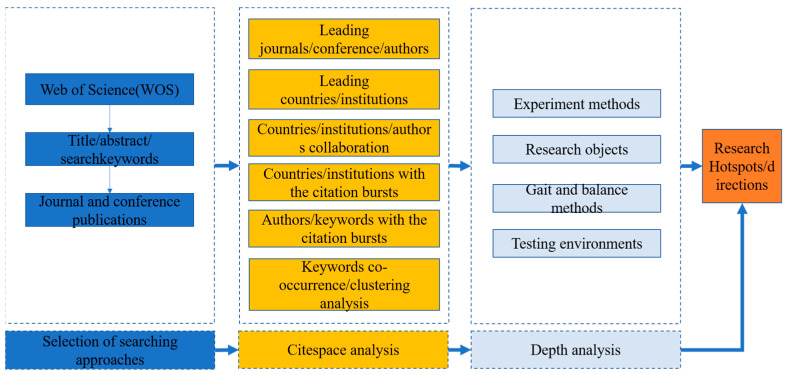
The flowchart of the review methods.

We selected the Web of Science (WoS) database for this investigation because it is renowned for its reputable and high-impact academic articles. A thorough search and analysis technique was necessary to ensure the calibre of the literature and enable speedy visualisation due to the abundance of publications on gait and balance. Thus, we restricted our search criteria to conferences and peer-reviewed journals only published in the English language. Figure 1 depicts the study’s overall structure. To analyse the literature retrieved from WoS, we used CiteSpace software (Version 6.2.2).

This study focuses on gait and balance-related journal and conference articles published between 1993 and 2020 in the scholarly database Web of Science (WoS). We conduct statistical analysis on academic journal trends, discipline distribution, journal distribution, research institution distribution, and research methodology using CiteSpace software. The keywords “older adults” and “gait and balance” were adopted as the selected criteria for the articles. In order to help researchers in the field, we aim to provide a thorough review of papers on gait and balance research, identifying research hotspots and development patterns. Our research provides a thorough grasp of the state of gait and balance studies today.

## 3. Visual Scientometric Analysis

### 3.1. Yearly Quantitative Analysis of Academic Publications

**Figure 2:**
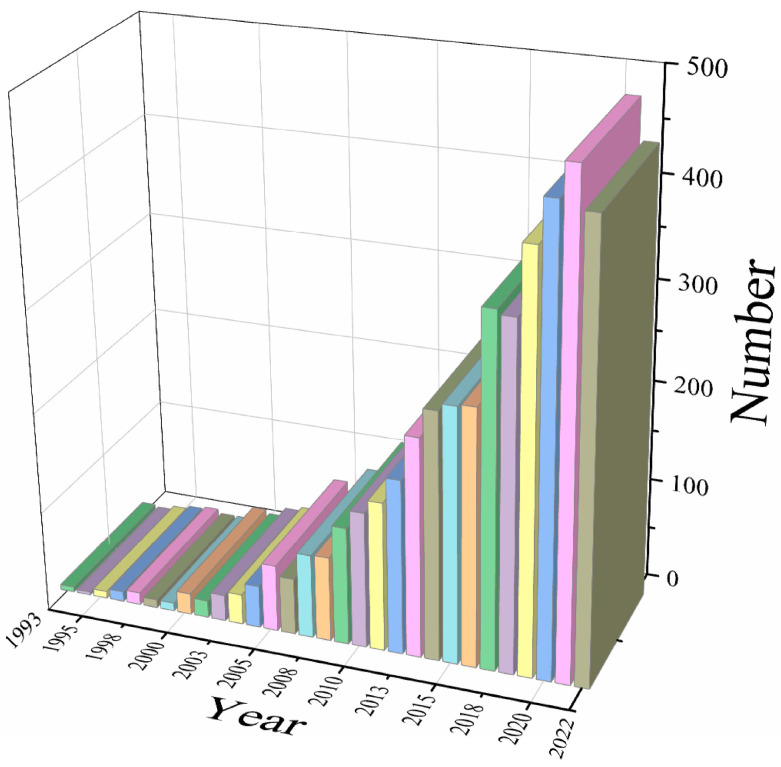
The change in academic publication numbers in gait and balance from 1993 to 2022.

As shown in Figure 2, 4484 academic publications including journal articles and conference proceedings on gait and balance in older adults from the year 1993 to 2022 are analysed.

The findings show a consistent rise in scholarly publications on gait and balance in senior citizens from 1993 to 2022, which reflects a persistent demand from the ageing population. Eight years stood out, with increases of 133.33%, 50%, 175%, 41.17%, 40%, 45.24%, 43.64%, 39.76%, and 38.91%, respectively. These years were 1995, 1997, 2000, 2006, 2007, 2009, and 2017. According to the data, there has been a steady increase between 1993 and 2021 in scientific papers about senior citizens’ gait and balance, which is a reflection of the population’s ageing-related demand. With gains of 133.33%, 50%, 175%, 41.17%, 40%, 45.24%, 43.64%, 39.76%, and 38.91%, respectively, eight years stood out. The years 1995, 1997, 2000, 2006, 2007, 2009, and 2017 were among them.

### 3.2. Timeline

In 1970, the field of gait and balance research started to take shape. The biomechanics of gait and balance as they relate to ageing, rehabilitation, and disease are the main topics of early research. [34,35]. In 1982, a method for measuring gait and balance objectively was devised [36], which serves as a starting point for the investigation of gait and balance in elderly people. In 1985, the deterioration of motor and sensory control processes emerged as a significant factor in falls [31]. In 1992, a study examining the impact of strength training and aerobic exercise on balance and gait in elderly male nursing home residents was conducted [37].

In 1993, research endeavoured to tackle several issues inherent in existing studies on exercise outcomes in older adults by employing an experimental design. Key features of the study design include a population-based sample; eligibility criteria based on physiological and functional deficits; random assignment to exercise groups; high-intensity exercise regimen; blinded assessment of physiological and functional outcomes; post-exercise follow-up; and a large sample size [38]. In 1995, the theory of lower limb strength and its relationship to gait and balance was put forth and researched [12]. In 2000, researchers used gait and balance to examine structural changes in the brains of men who had previously abstained from alcohol but later relapsed. The results revealed that men with alcoholism who maintain sobriety can show significant functional improvement that is connected to a change in brain structure [39]. In 2001, a dynamic model of ageing and illness that takes into account gait and balance issues was created [7]. Consequently, modern imaging and neurophysiology provide a more precise diagnosis and shed light on the pathogenesis of movement disorders [7]. Higher MRI white matter hyperintensity was thought to correlate with altered gait and balance in 2003 [10]. KineAssist, a robotic ground gait and balance training apparatus, was created and unveiled in 2005 [6]. In 2007, the impact of training regimens on healthy older adults’ gait and balance function was examined, and the theory has significant implications for enhancing older individuals’ gait and balance [40]. In 2010, there was increasing focus on techniques to improve gait and balance in Parkinson’s patients thanks to a comparison of the effects of partnered and unpartnered dance exercises on these two symptoms [41]. In 2011, research was conducted on the ground-based gait and balance training system ZeroG, which is crucial for the systematic observation and restoration of gait and balance [19].

In 2014, a study sought to quantitatively evaluate the impact of vigorous and light physical activity (VPA, LPA) on static balance, gait, and sit-to-stand (STS) tasks among a group of healthy older adults. Findings indicate significant improvements in most gait parameters and STS time within the VPA group, contrasting with the LPA group where such improvements were not observed [42]. Parkinson’s disease subtypes’ gait and balance are also being objectively measured and classified, which has significant consequences for the rehabilitation of patients with Parkinson’s [28]. The thorough analysis of traditional Chinese exercise’s impact on gait and balance in stroke patients demonstrates the significance of traditional Chinese exercise in the field of gait and balance research in 2015 [43]. In 2016, researchers looked into a measurement instrument for evaluating balance, gait, and posture in persons with Parkinson’s disease [13]. In 2017, the effect of small vessel disease in the brain on gait and balance was hypothesized and examined. The results showed that a variety of factors can affect gait and balance [44]. In 2019, research delved into quantifying the impacts of diverse factors on gait stability among older adults. The aim was to formulate tailored intervention strategies aimed at enhancing gait stability [45]. The year 2020 saw the proposal and implementation of virtual reality technology for gait and balance research and rehabilitation in Parkinson’s disease, which is crucial for the recovery and training of gait and balance in patients with Parkinson’s [8]. In 2022, a new study focus will be formed by the effects of remote gait and balance evaluation on studies conducted during and after COVID-19 [46].

The historical summary of seminal research on gait and balance demonstrates that the evaluation approach has been improved over time by the development of new techniques and models. Since the chronological summary lacks accurate statistics of significant study content, an analysis of keywords will be conducted to provide further information in the next section.

### 3.3. Leading Journals and Conference Proceedings

A rapid overview of the research landscape on the topic can be obtained by identifying top journals and conference proceedings. The top publications and conference proceedings for gait and balance research from 1993 through 2022 are shown in Tables S1 and S2, respectively. The top three journals are Gait Posture, Journals of Gerontology Series A Biological Sciences and Medical Sciences, and Journal of Biomechanics, while the top three conference proceedings are IEEE Engineering in Medicine and Biology Society Conference Proceedings, 2011 Annual International Conference of the IEEE Engineering in Medicine and Biology Society (EMBC), and Gerontologist. The findings show that publications on gait and balance studies were primarily focused on medical and rehabilitation fields. To fill in any gaps in the study information in this subsection, additional keyword analysis will be conducted.

### 3.4. Keywords

**Figure 3:**
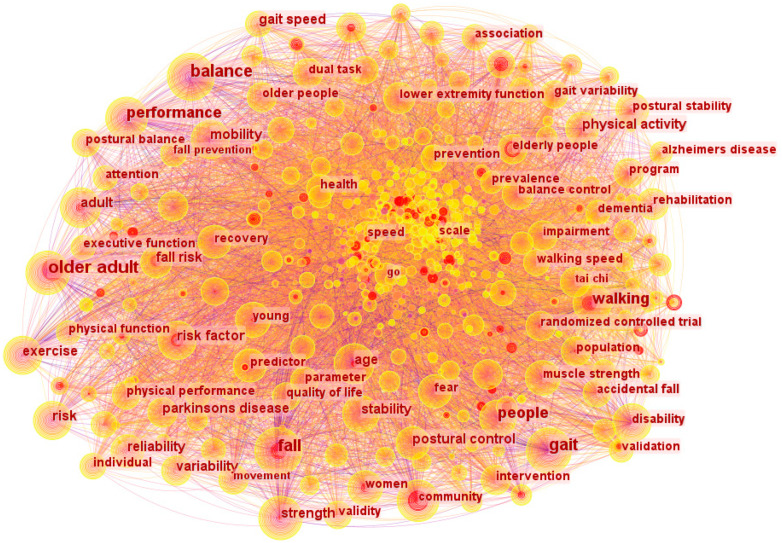
Keyword distribution analysis.

A keyword analysis is shown in Figure 3 to acquire a more thorough grasp of the important ideas and developments in gait and balance research and development.

The analysis results indicate that “older adults”, “balance”, “gait”, and “fall” are the keywords that co-occur most frequently. The earliest occurrences were of the terms “balance”, “fall”, and “gait”. The keyword “older adults” appears most frequently. The radius of the red circle represents the frequency of occurrence. The study of balance among older adults encompasses the analysis of falls and their associated risks, as well as prediction models. Balance is affected by factors such as stability, strength, and exercise. It shows the research has explored the balance change with age or injury, and the approach to improving them can enhance the quality of life and reduce the risk of falls.

**Figure 4:**
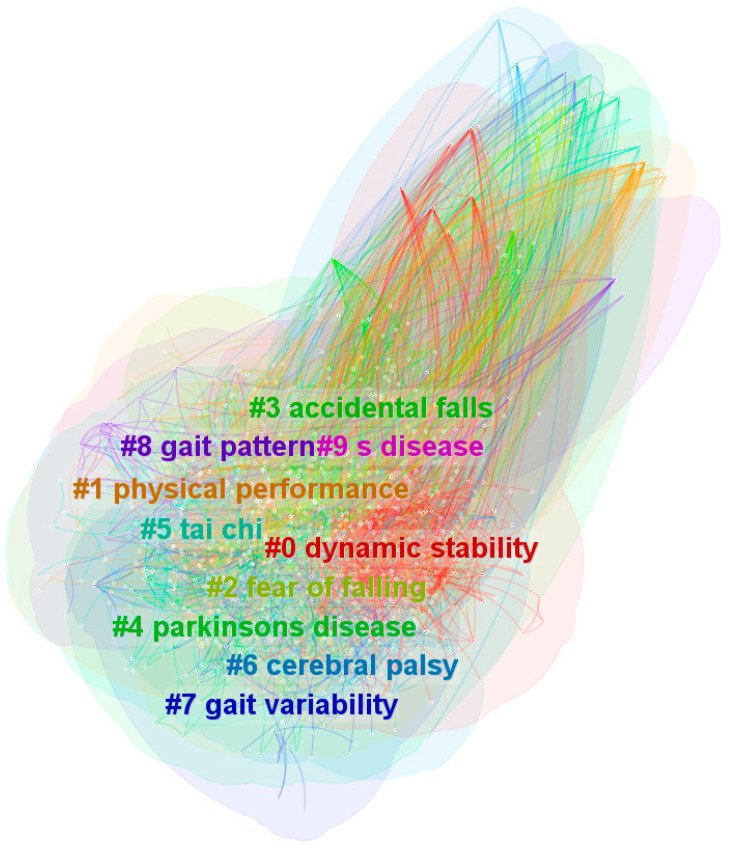
Clustering for the keywords.

To further investigate the cluster analysis of the keywords, we identified a total of nine clusters. The clusters, based on keyword frequency, are displayed in different colours in Figure 4. The cluster analysis algorithm used was LLR (Log-Likelihood Rate).

Figure 4 demonstrates that clustering provides a more accurate classification of research directions and hotspots in the field. Two indicators commonly used to assess the effectiveness of cluster analysis are modularity and silhouette. An index exceeding 0.3 is often used to determine whether the structure of a cluster analysis is reasonable. Figure 4 achieved modularity and silhouette values of 0.3474 and 0.6668, respectively, indicating a high level of reliability in the cluster analysis. The categories are dynamic stability, physical performance, fear of falling, accidental falls, Parkinson’s disease, tai chi, cerebral palsy, gait variability, gait pattern, and disease. Numbers #0–#9 correspond to the frequencies of the keywords, ranked from highest (#0) to lowest (#9). The clusters are ranked from #0 to #9, with #0 focusing on dynamic stability as the core research component dating back to 2007, with topics including postural control, perturbation, risk factors, and parameter estimation. Cluster #1, physical performance, includes topics such as nursing homes, controlled trials, multicomponent exercise programmes, and physical activity, with an average year of 2008. Cluster #2, fear of falling, includes topics such as postural balance, exercise therapy, Parkinson’s disease, and neurological rehabilitation, with a new trend of combining artificial neural networks and dual extended Kalman filters. Cluster #3, accidental falls, focuses on postural control, risk factors, Alzheimer’s disease, and various methods and technologies for assessing and enhancing gait and balance, including wearable sensors, virtual reality training, and exercise programs. Cluster #4, Parkinson’s disease required physical education, including topics such as mobility performance, smartphones, clinical measures, and performance evaluation approaches focusing on motion-capturing systems and virtual reality, with the average year of 2012. Cluster #5, tai chi research, includes topics such as fall prevention, multiple sclerosis, and Xbox Kinect, with an average year of 2010. Cluster #6, cerebral palsy, includes topics such as diabetes insipidus, palpebral fissure, glucose tolerance, and dexamethasone suppression. Cluster #7, gait variability research, focuses on diabetes insipidus, palpebral fissure, glucose tolerance, and the common approach of parameter estimation by inertial sensors. Cluster #8, gait pattern, includes topics such as risk of falling and bone mineral density. Finally, Cluster #9 indicates diseases occurring in dwelling older adults, with topics including Parkinson’s disease, mild cognitive impairment, and neurological disorders. Table 1 demonstrates that older adults in the community have been the primary subjects of research in the field of gait and balance. The researchers also paid attention to motility and lower extremity strength.

### 3.5. Country

**Figure 5:**
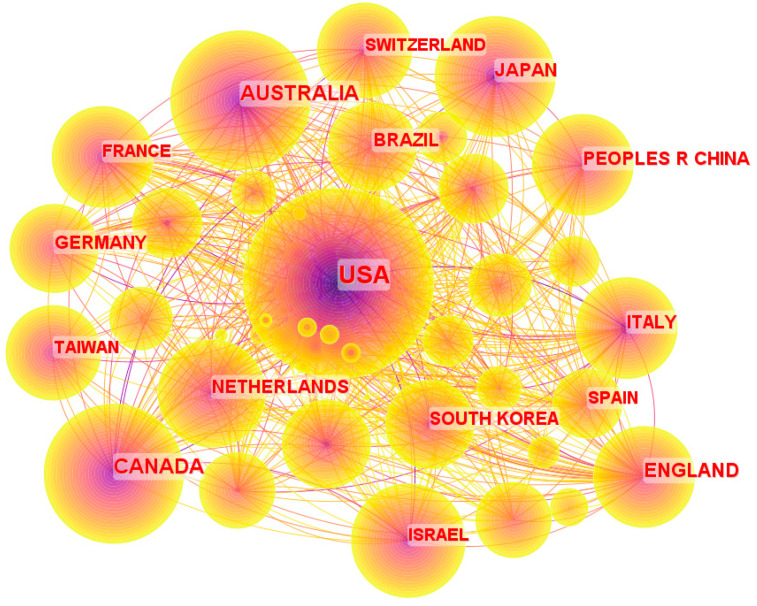
The distribution map of countries.

Figure 5 depicts the geographical distribution of the published literature on gait and balance, with 80 nodes representing 80 countries and the circular radius of each node representing the number of publications. The United States (US) exhibits the highest number of publications with 1742 articles, followed by Canada (432 articles), Australia (344 articles), and England (279 articles). These countries have demonstrated a significant contribution to the advancement of the field of gait and balance through their extensive research output. It is worth noting that other nations, although not explicitly highlighted in the graph, have also made noteworthy contributions in this area.

A valuable tool for analysing the frequency of publications in a certain nation over a given time period is the citation burst index, which offers information on the trends and advancements of nations, organisations, and keywords in the field of gait and balance. The top 6 nations with the most significant citation bursts are shown in Table 2, showing their significance and contributions to the discipline. Researchers and practitioners can use this study to stay current on the most recent research developments and to find possible areas for collaboration and knowledge exchange.

The citation burst index serves as a benchmark for trends and developments in the gait and balance research community by revealing information about the frequency of publications in a certain nation during a given period of time. By frequently published articles, the six nations with the highest citation burst index have shaped gait and balance research. Importantly, as seen by their impressive citation burst indices of 44.56 and 19.52, respectively, the USA and Canada have been leaders in gait and balance research for a considerable amount of time. Since 2019, Spain and the Czech Republic have made recent strides in this industry. With a citation burst index of 7.48, Switzerland comes in third place, demonstrating its research potential and competitiveness in the field of gait and balance. With information on chronology and burst strength paired with a country-by-country examination of gait and balance research, academic collaboration and communication among academics are encouraged.

### 3.6. Author

**Figure 6:**
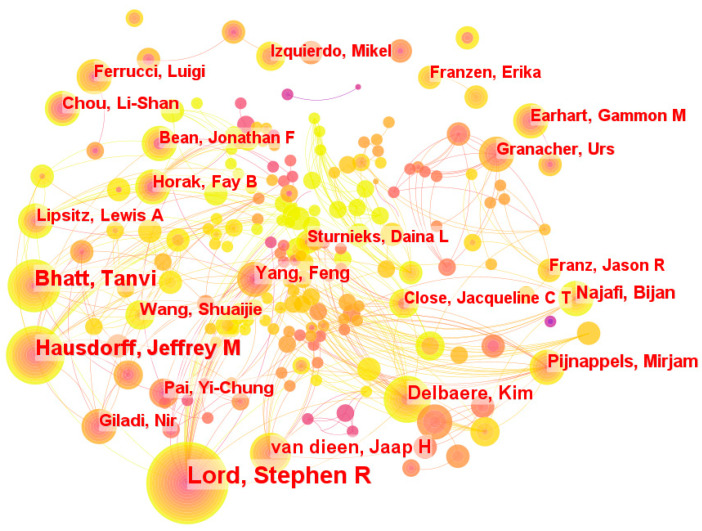
The distribution map of the authors.

Stephen R. Lord is the author of 61 articles and is the author with the most publications on gait and balance. Indicating the importance and relevance of their most recent research advancement, the burst coefficients of Wang, Shuaijie, and Franzen, Erika, were discovered to be as high as 6.39 and 6.48, respectively, between 2016 and 2020. Also, Hausdorff, Jeffrey My has shown the ground-breaking relevance of their team’s research in the area of gait and balance with a total of 35 published publications and a burst coefficient of 3.67 between 2013 and 2016. These scholars’ contributions have increased our understanding of gait and balance, and their work offers valuable insights and recommendations for further study in the area. See Figure 6.

**Figure 7:**
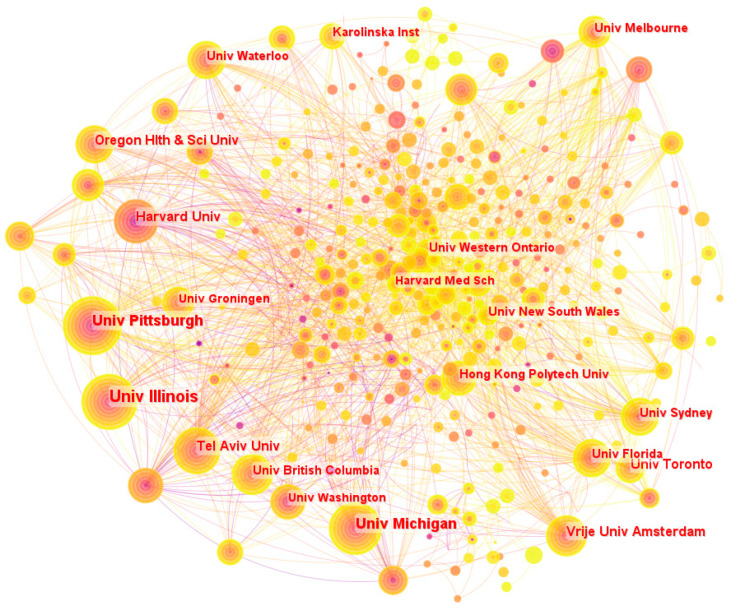
The distribution map of universities.

The analysis from Figure 7 shows that the University of Pittsburgh, which has a total of 103 papers with a burst factor of 7.7, is at the forefront of gait and balance research. With a burst factor of 20.46, Harvard University, on the other hand, has made impressive strides in the subject, demonstrating its dedication to enhancing gait and balance research. With a combined total of 50, 71, and 53 articles, respectively, Tel Aviv University, the University of British Columbia, and the Hong Kong Polytechnic University have made major contributions to this topic. When it comes to finding possible research partners and encouraging global collaboration to further scientific discovery and innovation, such information is helpful to the academic community.

## 4. Research Approaches for Gait and Balance

Several disciplines, including neuroscience, rehabilitation, sports medicine, and biomechanics, have shown an interest in the study of gait and balance [47,48,49]. Understanding how people move while remaining stable and reducing their risk of falling is the goal of the gait and balance field of study [3,50,51,52]. The mechanics of gait and balance have been studied using a variety of study approaches, including experimental studies, observational studies, and computational modelling.

### 4.1. Experimental Studies

To determine how particular parameters affect gait and balance, experimental research directly manipulates variables such as changes in body weight distribution, visual cues, and outside disturbances [37,43,53,54,55,56]. Instrumented treadmills, force plates, motion capture systems, and other pieces of equipment are frequently used in this research to monitor a variety of variables, including joint angles, ground response forces, and muscle activity [29,39,57,58,59,60,61,62,63]. Intervention studies that use exercise regimens, physical therapy, or other forms of care to enhance gait and balance in people with particular medical disorders are also considered experimental investigations [2,64,65,66,67,68,69].

### 4.2. Observational Studies

Conversely, observational studies concentrate on monitoring and evaluating gait and balance in various populations, such as children, the elderly, and those with neurological diseases, rather than directly manipulating parameters [12,70,71,72,73]. These studies can evaluate gait and balance features, such as stride length, gait speed, and postural sway, using a variety of observational techniques, including clinical scales, video recordings, and wearable sensors [74,75,76,77,78,79,80].

### 4.3. Computational Modelling

In contrast, observational studies concentrate on observing and analysing gait and balance in various populations, such as children, the elderly, and people with neurological disorders [81,82,83,84,85]. These studies do not directly manipulate parameters, instead focusing on gait and balance in different populations. To evaluate gait and balance features such as stride length, gait speed, and postural sway, these studies can use a variety of observational techniques, including clinical scales, video recordings, and wearable sensors [86,87,88,89,90,91,92,93,94].

Wearable devices, including accelerometers, gyroscopes, and pressure sensors, have been developed as a result of contemporary technical advancements, and they can be used to monitor balance and gait when outside [94,95,96,97]. These devices can be combined with machine-learning algorithms to assess enormous amounts of data and discover patterns that may be useful for recognising and treating gait and balance disorders [98,99,100]. Virtual reality (VR) technology has also been used to create immersive environments that may mimic a range of real-world situations as well as gait and balance problems [101,102,103]. VR can be used to study how various environmental factors affect gait and balance or as a training tool to help persons with mobility issues walk and balance better.

In conclusion, research on gait and balance involves various approaches, including experimental studies, observational studies, and computational modelling, as well as the use of wearable devices and VR technology. These approaches have significantly enhanced our understanding of the mechanisms underlying gait and balance control, resulting in new insights into the diagnosis and treatment of related disorders. Future research in this field is expected to delve deeper into emerging technologies and methodologies to advance our knowledge of gait and balance control.

## 5. Research Contents of Gait and Balance

### 5.1. Participants

Studies on gait and balance often focus on older individuals due to their increased susceptibility to age-related changes in these bodily systems [104,105,106]. Participants are often considered “older” if they are over the age of 65 [107,108,109]. The musculoskeletal, neurological, and sensory systems are more likely to shift in this age range, which might affect balance and gait [109]. Age-related changes in muscular strength, joint flexibility, and sensory perception, for instance, might alter gait patterns and increase the risk of falling in older persons [110,111,112,113,114].

Because older persons are more prone to experience age-related alterations in their physical systems, older adults are commonly utilised as study subjects in gait and balance research [115,116,117]. If a participant is over the age of 65, they are frequently seen as “older” [117,118,119]. At this age range, the musculoskeletal, neurological, and sensory systems are more likely to change, which could have an impact on balance and gait [119,120,121,122]. For example, age-related changes in sensory perception, joint flexibility, and muscle strength may modify gait patterns in older people and increase their risk of falling [119,123,124,125].

Nervous system disorders like Parkinson’s disease, multiple sclerosis, and cerebral palsy are among the neurological illnesses that frequently affect study participants in gait and balance [126,127,128]. Changes in gait and balance can occur as a result of these disorders having an impact on the neurological system [129,130,131]. For instance, stiffness, bradykinesia, and tremors are symptoms of Parkinson’s disease that can affect a person’s walk and balance. Knowing how these individuals’ gaits and balances vary can help guide therapies that could increase their mobility and lower their risk of falling [132,133,134,135].

Another participant group that can be researched in gait and balance studies is athletes, especially in sports that demand agility, coordination, and balance [136,137,138,139]. Compared to elderly persons and people with neurological disorders, athletes are often younger and have different physical traits [77,139,140]. They might, for instance, have more flexible and strong muscles, which can affect the way they walk and how they balance. Knowing an athlete’s gait and balance patterns might help develop training plans that could boost their performance and lower their risk of injury [140].

Children are a participant group in studies on gait and balance as well, particularly those who seek to understand how these abilities develop. Children’s musculoskeletal, neurological, and sensory systems alter as they grow and develop, which affects how they walk and balance [141,142]. Interventions that could enhance their motor abilities and lower their risk of falling can be informed by an understanding of these developments [143]. To comprehend how these disorders affect gait and balance, research may also be conducted on kids with developmental disabilities, such as cerebral palsy.

The research question, study design, and sample size are only a few of the variables that affect how participants are chosen in gait and balance studies [144]. For instance, older folks would be the ideal participant group if the study’s goal was to examine the efficiency of a gait training programme in this population. The choice of participants may also be influenced by the study’s design, such as a randomised controlled trial. The sample size should be adequate to identify significant changes in these functions, for instance, if the study’s goal is to examine how an intervention affects gait and balance.

To comprehend the elements that affect these processes, it is crucial to carefully choose volunteers for gait and balance studies. Gait and balance study frequently examines older adults, those with neurological disorders, athletes, and children, each of whom offers a different perspective on the influences of these processes [137,145]. A number of variables, such as the research topic, study design, and sample size, have an impact on participant selection [139]. Designing studies that offer useful insights into gait and balance requires an understanding of participant characteristics and the factors that affect participant selection.

### 5.2. Environments

The qualities of the surface on which gait and balancing exercises are carried out are referred to as surface characteristics [139,146]. Surface qualities, including roughness, hardness, and slipperiness, can have a big effect on how a participant walks and balances [147]. Walking on a damp or slippery surface, for instance, might lead to instability and alter gait patterns. When choosing surfaces for their studies, researchers should take into account the environment’s surface characteristics.

Gait and balance performance can also be affected by lighting conditions [148]. Inadequate lighting can make falls more likely and make gait and balance assessments less accurate. The testing environment’s illumination should be suitable for the tasks being carried out, and participants should be able to view the testing equipment properly. A participant’s ability to focus on the job at hand may be impacted by noise in the testing area, which can be distracting. In order to ensure that participants can concentrate on the gait and balance task without being distracted, researchers should keep testing environments as quiet as possible.

Another crucial aspect of gait and balance is the ambient temperature [140]. Excessive temperatures can make people tired and alter their walking patterns. For participants to complete the activity safely and accurately, the testing area should be at a comfortable temperature, according to researchers.

Spatial limitations have always been important when evaluating participants’ gait and balance [149]. A participant’s ability to complete some gait and balance activities may be restricted in small testing areas, but fatigue and longer walking distances may develop in larger testing regions. While choosing a suitable testing space that enables participants to complete the job safely and accurately, researchers should take into account the physical limits of the environment.

Researchers should carefully take into account the surface qualities, illumination, noise, temperature, and space limits as these environmental elements may have an impact on participant performance. By taking into account these variables, researchers can guarantee the validity and reliability of their study findings as well as the safety of test subjects.

## 6. Discussion and Limitations

Gait and balance are crucial aspects of daily life, and problems with these abilities can cause falls, injuries, and a decline in quality of life, particularly in senior people. By leveraging the WoS database, this study seeks to examine the body of knowledge, research horizons, and application trends of gait and balance. Correlation analysis and CiteSpace processing of the literature were used in the research technique. Gait and balance research hotspots were analysed using the co-citation theory and burst detection analysis. This study seeks to provide a clear and intuitive observation of the development path and research trend of gait and balance through visualisation of the research and analysis. This method makes a significant addition to the academic community by giving a thorough grasp of the gait and balance research environment.

(1) Gait and balance research usually refers to “older individuals” and “balance”, which suggests that older persons have been the primary research subjects for studies of gait and balance as shown in Figure 3. The experimental sample was primarily composed of elderly residents of the community who were not receiving any care.
(2) The analysis and characterisation of experimental results in the area of gait and balance depend heavily on physical performance evaluation. Significantly, as seen in Figure 4, research on particular populations and disorders, like Alzheimer’s disease, is centred in two main groups, namely Cluster #1 and Cluster #9.
(3) A significant portion of the keyword analysis is devoted to the research of gait speed and walking speed, as illustrated in Figure 3, and speed in balance is a crucial evaluation criterion.
(4) Figure 3 shows that the terms fall prevention, posture control, etc. are commonly used in keyword searches, thus it is important to prioritise posture control research using different tools like virtual reality, artificial intelligence algorithms, wearable sensors, etc.

Despite the increased interest in gait and balance research, the terms gait and balance are not consistently defined, which makes it challenging to compare study findings and prevents the area from developing a common vocabulary. To comprehend how gait and balance problems develop over time, further longitudinal research in the field is required. Also, more study is required to determine how exercises and physical therapy affect clinical groups’ gait and balance. An interdisciplinary strategy involving cooperation between researchers from several domains, including biomechanics, neuroscience, and rehabilitation, is necessary due to the intricacy of gait and balance. The compartmentalised structure of many research institutes, however, can restrict collaboration and impede advancement in the area.

## 7. Conclusions

A rapidly expanding subject, gait and balance research has significant implications for therapeutic practise. Gait and balance research has recently concentrated on a few trends. There is a growing trend towards employing technology to analyse and improve gait and balance. Recent studies have evaluated and trained gait and balance using wearable sensors, virtual reality, and artificial intelligence algorithms [147,148,149]. Furthermore, there has been a surge of interest among researchers to investigate the effects of exercise on gait and balance. This growing body of research aims to shed light on the mechanisms underlying the observed improvements in gait and balance among different populations, such as older adults or individuals with neurological disorders, and to optimize exercise interventions that promote functional mobility and reduce fall risk. Gait and balance can be improved by exercise regimens in both healthy and clinical populations. The impact of cognitive and sensory deficits on gait and balance has drawn more attention in recent years. It has been established through research that therapies aimed at improving cognitive and sensory deficits can also enhance gait and balance. Research on how environmental influences affect gait and balance is becoming more and more popular. According to research, gait and balance can be impacted by ambient elements like sunlight, surface properties, and impediments. In order to enhance the health and wellbeing of people with gait and balance impairments, clinicians and researchers should take these patterns into account when developing treatment plans and research projects.

The path of the research on gait and balance is becoming more entangled with computer science and artificial intelligence (AI). Intelligent gait balance monitoring for the elderly is poised to emerge as a prominent area of research in the foreseeable future, drawing significant attention from scholars and researchers alike. Future directions in gait and balance research are expected to emphasize multidisciplinary collaboration, intelligence, and visualization.

## Figures and Tables

**Table 1:** The top 30 keywords with the biggest bursts of citations (Blue boxes show the period without a burst, and red boxes represent the burst period from beginning to end).

Keywords	Year	Strength	Begin	End	1993–2023
Elderly person	1995	18.04	1995	2014	▂▂ ▃▃▃▃▃▃▃▃▃▃▃▃▃▃▃▃▃▃▃▃ ▂▂▂▂▂▂▂▂▂
Community	1997	16.12	1997	2013	▂▂▂▂ ▃▃▃▃▃▃▃▃▃▃▃▃▃▃▃▃▃ ▂▂▂▂▂▂▂▂▂▂
Women	1996	12.85	2003	2011	▂▂▂ ▂▂▂▂▂▂▂ ▃▃▃▃▃▃▃▃▃ ▂▂▂▂▂▂▂▂▂▂▂▂
Age	1993	9.96	1996	2009	▂▂▂ ▃▃▃▃▃▃▃▃▃▃▃▃▃▃ ▂▂▂▂▂▂▂▂▂▂▂▂▂▂
Locomotion	1997	9.66	1997	2014	▂▂▂▂ ▃▃▃▃▃▃▃▃▃▃▃▃▃▃▃▃▃▃ ▂▂▂▂▂▂▂▂▂
Men	1993	9.04	1993	2010	▃▃▃▃▃▃▃▃▃▃▃▃▃▃▃▃▃▃ ▂▂▂▂▂▂▂▂▂▂▂▂▂
Posturography	2000	9.03	2000	2011	▂▂▂▂▂▂▂ ▃▃▃▃▃▃▃▃▃▃▃▃ ▂▂▂▂▂▂▂▂▂▂▂▂
Strength	1998	8.31	1998	2006	▂▂▂▂▂ ▃▃▃▃▃▃▃▃▃ ▂▂▂▂▂▂▂▂▂▂▂▂▂▂▂▂▂
Tai chi	2004	7.29	2004	2008	▂▂▂▂▂▂▂▂▂▂▂ ▃▃▃▃▃ ▂▂▂▂▂▂▂▂▂▂▂▂▂▂▂
Rating scale	2010	6.96	2010	2016	▂▂▂▂▂▂▂▂▂▂▂▂▂▂▂▂▂ ▃▃▃▃▃▃▃ ▂▂▂▂▂▂▂
Human	1996	6.88	1996	2007	▂▂▂ ▃▃▃▃▃▃▃▃▃▃▃▃ ▂▂▂▂▂▂▂▂▂▂▂▂▂▂▂▂
Exercise	1995	6.88	1995	2004	▂▂ ▃▃▃▃▃▃▃▃▃▃ ▂▂▂▂▂▂▂▂▂▂▂▂▂▂▂▂▂▂▂
Elderly patient	1996	6.76	1996	2005	▂▂▂ ▃▃▃▃▃▃▃▃▃▃ ▂▂▂▂▂▂▂▂▂▂▂▂▂▂▂▂▂▂
Randomized controlled trial	1999	6.66	2014	2018	▂▂▂▂▂▂ ▂▂▂▂▂▂▂▂▂▂▂▂▂▂▂ ▃▃▃▃▃ ▂▂▂▂▂
Machine learning	2021	6.61	2021	2023	▂▂▂▂▂▂▂▂▂▂▂▂▂▂▂▂▂▂▂▂▂▂▂▂▂▂▂▂ ▃▃▃
Balance control	1996	6.31	2006	2008	▂▂▂ ▂▂▂▂▂▂▂▂▂▂ ▃▃▃ ▂▂▂▂▂▂▂▂▂▂▂▂▂▂▂
Hip fracture	1995	6.26	1995	2009	▂▂ ▃▃▃▃▃▃▃▃▃▃▃▃▃▃▃ ▂▂▂▂▂▂▂▂▂▂▂▂▂▂
Accelerometer	2015	6.22	2015	2018	▂▂▂▂▂▂▂▂▂▂▂▂▂▂▂▂▂▂▂▂▂▂ ▃▃▃▃ ▂▂▂▂▂
Dwelling older adult	2002	6.11	2012	2017	▂▂▂▂▂▂▂▂▂ ▂▂▂▂▂▂▂▂▂▂ ▃▃▃▃▃▃ ▂▂▂▂▂▂
Postural response	2003	6.07	2003	2009	▂▂▂▂▂▂▂▂▂▂ ▃▃▃▃▃▃▃ ▂▂▂▂▂▂▂▂▂▂▂▂▂▂
Quality	2004	5.98	2018	2023	▂▂▂▂▂▂▂▂▂▂▂ ▂▂▂▂▂▂▂▂▂▂▂▂▂▂ ▃▃▃▃▃▃
Cognitive impairment	2010	5.93	2019	2023	▂▂▂▂▂▂▂▂▂▂▂▂▂▂▂▂▂ ▂▂▂▂▂▂▂▂▂ ▃▃▃▃▃
Sway	2002	5.87	2002	2011	▂▂▂▂▂▂▂▂▂ ▃▃▃▃▃▃▃▃▃▃ ▂▂▂▂▂▂▂▂▂▂▂▂
Gait initiation	2007	5.79	2007	2013	▂▂▂▂▂▂▂▂▂▂▂▂▂▂ ▃▃▃▃▃▃▃ ▂▂▂▂▂▂▂▂▂▂
Motor	2005	5.68	2020	2023	▂▂▂▂▂▂▂▂▂▂▂▂ ▂▂▂▂▂▂▂▂▂▂▂▂▂▂▂ ▃▃▃▃
Mini BEStest	2017	5.58	2017	2021	▂▂▂▂▂▂▂▂▂▂▂▂▂▂▂▂▂▂▂▂▂▂▂▂ ▃▃▃▃▃ ▂▂
Meta-analysis	2005	5.57	2017	2018	▂▂▂▂▂▂▂▂▂▂▂▂ ▂▂▂▂▂▂▂▂▂▂▂▂ ▃▃ ▂▂▂▂▂
Pattern	1997	5.52	2005	2011	▂▂▂▂ ▂▂▂▂▂▂▂▂ ▃▃▃▃▃▃▃ ▂▂▂▂▂▂▂▂▂▂▂▂
Rehabilitation	2004	5.5	2009	2014	▂▂▂▂▂▂▂▂▂▂▂ ▂▂▂▂▂ ▃▃▃▃▃▃ ▂▂▂▂▂▂▂▂▂
Adult	1993	5.47	2000	2009	▂▂▂▂▂▂▂ ▃▃▃▃▃▃▃▃▃▃ ▂▂▂▂▂▂▂▂▂▂▂▂▂

**Table 2:** Top six countries with the strongest citation bursts (Blue boxes show the period without a burst, and red boxes represent the burst period from beginning to end).

Countries	Year	Strength	Begin	End	1993–2022
USA	1993	44.56	1993	2007	▃▃▃▃▃▃▃▃▃▃▃▃▃▃▃ ▂▂▂▂▂▂▂▂▂▂▂▂▂▂▂
Spain	2010	10.26	2019	2022	▂▂▂▂▂▂▂▂▂▂▂▂▂▂▂▂▂ ▂▂▂▂▂▂▂▂▂ ▃▃▃▃
Switzerland	2001	7.48	2010	2013	▂▂▂▂▂▂▂▂ ▂▂▂▂▂▂▂▂▂ ▃▃▃▃ ▂▂▂▂▂▂▂▂▂
Canada	1994	4.92	2002	2007	▂ ▂▂▂▂▂▂▂▂ ▃▃▃▃▃▃ ▂▂▂▂▂▂▂▂▂▂▂▂▂▂▂
Saudi Arabia	2013	3.82	2014	2015	▂▂▂▂▂▂▂▂▂▂▂▂▂▂▂▂▂▂▂▂ ▂ ▃▃ ▂▂▂▂▂▂▂
Czech Republic	2008	3.71	2020	2022	▂▂▂▂▂▂▂▂▂▂▂▂▂▂▂ ▂▂▂▂▂▂▂▂▂▂▂▂ ▃▃▃

## Data Availability

Data are available upon readers’ request.

## Supplementary Materials

The following supporting information can be downloaded at: https://www.mdpi.com/article/10.3390/s24103199/s1, Table S1: Leading Journal Publications in gait and balance from 1993 to 2022; Table S2: Leading Conference Publications in gait and balance from 1993 to 2022.
