# Supplementary material for: Scientometric Research and Critical Analysis of Gait and Balance in Older Adults
Source: Sensors (Basel). 2024 May 17;24(10):3199. doi: 10.3390/s24103199 (PMC11125350; doi:10.3390/s24103199)
Supplement: Supplementary file 1 [file sensors-24-03199-s001.zip › Table S1.pdf]

Table S1 Leading Journal Publications in gait and balance from 1993 to 2022

Journal title	Number of articles	%Total publications
GAIT POSTURE	499	11.53
JOURNALS OF GERONTOLOGY SERIES A BIOLOGICAL SCIENCES AND MEDICAL SCIENCES	114	2.634
JOURNAL OF BIOMECHANICS	93	2.149
ARCHIVES OF PHYSICAL MEDICINE AND REHABILITATION	91	2.103
BMC GERIATRICS	90	2.079
JOURNAL OF THE AMERICAN GERIATRICS SOCIETY	86	1.987
AGING CLINICAL AND EXPERIMENTAL RESEARCH	79	1.825
JOURNAL OF GERIATRIC PHYSICAL THERAPY	71	1.64
PLOS ONE	71	1.64
PHYSICAL THERAPY	69	1.594
INTERNATIONAL JOURNAL OF ENVIRONMENTAL RESEARCH AND PUBLIC HEALTH	68	1.571
JOURNAL OF AGING AND PHYSICAL ACTIVITY	66	1.525

SENSORS	66	1.525
ARCHIVES OF GERONTOLOGY AND GERIATRICS	58	1.34
EXPERIMENTAL BRAIN RESEARCH	53	1.225
EXPERIMENTAL GERONTOLOGY	53	1.225
FRONTIERS IN AGING NEUROSCIENCE	53	1.225
CLINICAL BIOMECHANICS	50	1.155
JOURNAL OF NEUROENGINEERING AND REHABILITATION	49	1.132
GERONTOLOGY	45	1.04
SCIENTIFIC REPORTS	42	0.97
HUMAN MOVEMENT SCIENCE	41	0.947
JOURNAL OF PHYSICAL THERAPY SCIENCE	41	0.947
AGE AND AGEING	39	0.901
CLINICAL REHABILITATION	39	0.901
DISABILITY AND REHABILITATION	39	0.901
CLINICAL INTERVENTIONS IN AGING	37	0.855

JOURNAL OF THE AMERICAN MEDICAL DIRECTORS ASSOCIATION	37	0.855
FRONTIERS IN NEUROLOGY	36	0.832
GERIATRICS GERONTOLOGY INTERNATIONAL	35	0.809
TOPICS IN GERIATRIC REHABILITATION	32	0.739
JOURNAL OF NUTRITION HEALTH AGING	29	0.67
AMERICAN JOURNAL OF PHYSICAL MEDICINE REHABILITATION	26	0.601
JOURNAL OF NEUROPHYSIOLOGY	25	0.578
PHYSIOTHERAPY THEORY AND PRACTICE	25	0.578
JOURNAL OF NEUROLOGIC PHYSICAL THERAPY	24	0.555
FRONTIERS IN HUMAN NEUROSCIENCE	22	0.508
JOURNAL OF MOTOR BEHAVIOR	22	0.508
NEUROREHABILITATION AND NEURAL REPAIR	22	0.508
EUROPEAN REVIEW OF AGING AND PHYSICAL ACTIVITY	21	0.485
BMC NEUROLOGY	19	0.439
MATURITAS	19	0.439

**BRAIN SCIENCES**	**17**	**0.393**
**JOURNAL OF CLINICAL MEDICINE**	**17**	**0.393**
**FRONTIERS IN MEDICINE**	**16**	**0.37**
**FRONTIERS IN SPORTS AND ACTIVE LIVING**	**16**	**0.37**
