# Supplementary material for: Scientometric Research and Critical Analysis of Gait and Balance in Older Adults
Source: Sensors (Basel). 2024 May 17;24(10):3199. doi: 10.3390/s24103199 (PMC11125350; doi:10.3390/s24103199)
Supplement: Supplementary file 1 [file sensors-24-03199-s001.zip › Table S2.pdf]

Table S2 Leading Conference Publications in gait and balance from 1993 to 2022

Conference title	Number of articles	%Total publications
IEEE ENGINEERING IN MEDICINE AND BIOLOGY SOCIETY CONFERENCE PROCEEDINGS	18	18.182
2011 ANNUAL INTERNATIONAL CONFERENCE OF THE IEEE ENGINEERING IN MEDICINE AND BIOLOGY SOCIETY EMBC	6	6.061
GERONTOLOGIST	6	6.061
MEDICINE AND SCIENCE IN SPORTS AND EXERCISE	5	5.051
IEEE INTERNATIONAL CONFERENCE ON SYSTEMS MAN AND CYBERNETICS CONFERENCE PROCEEDINGS	4	4.040
JOURNAL OF THE AMERICAN GERIATRICS SOCIETY	4	4.040
LECTURE NOTES IN COMPUTER SCIENCE	4	4.040
2013 35TH ANNUAL INTERNATIONAL CONFERENCE OF THE IEEE ENGINEERING IN MEDICINE AND BIOLOGY SOCIETY EMBC	3	3.030
2021 43RD ANNUAL INTERNATIONAL CONFERENCE OF THE IEEE ENGINEERING IN MEDICINE BIOLOGY SOCIETY EMBC	3	3.030
INTERNATIONAL CONFERENCE ON REHABILITATION ROBOTICS ICORR	3	3.030
2016 38TH ANNUAL INTERNATIONAL CONFERENCE OF THE IEEE ENGINEERING IN MEDICINE AND BIOLOGY SOCIETY EMBC	2	2.020
ASSISTIVE TECHNOLOGY RESEARCH SERIES	2	2.020
IEEE INTERNATIONAL SYMPOSIUM ON MEDICAL MEASUREMENTS AND APPLICATIONS PROCEEDINGS MEMEA	2	2.020
INTERNATIONAL CONGRESS SERIES	2	2.020
JOURNAL OF AGING AND PHYSICAL ACTIVITY	2	2.020
VESTIBULAR AND NEURAL FRONT	2	2.020
11TH IEEE SYMPOSIUM ON COMPUTER APPLICATIONS INDUSTRIAL ELECTRONICS ISCAIE 2021	1	1.010
12TH INTERNATIONAL CONFERENCE ON KINANTHROPOLOGY	1	1.010
2006 28TH ANNUAL INTERNATIONAL CONFERENCE OF THE IEEE ENGINEERING IN MEDICINE AND BIOLOGY SOCIETY VOLS 1 15	1	1.010
2006 IEEE INTERNATIONAL CONFERENCE ON SYSTEMS MAN AND CYBERNETICS VOLS 1 6 PROCEEDINGS	1	1.010
2007 IEEE INTERNATIONAL CONFERENCE ON SYSTEMS MAN AND CYBERNETICS VOLS 1 8	1	1.010
2007 VIRTUAL REHABILITATION	1	1.010
2008 IEEE INTERNATIONAL CONFERENCE ON SYSTEMS MAN AND CYBERNETICS SMC VOLS 1 6	1	1.010
2009 VIRTUAL REHABILITATION INTERNATIONAL CONFERENCE	1	1.010

**2010 ANNUAL INTERNATIONAL CONFERENCE OF THE IEEE ENGINEERING IN MEDICINE AND BIOLOGY SOCIETY EMBC**	**1**	**1.010**
**2011 INTERNATIONAL CONFERENCE OF ENVIRONMENTAL SCIENCE AND ENGINEERING VOL 12 PT B**	**1**	**1.010**
**2012 ANNUAL INTERNATIONAL CONFERENCE OF THE IEEE ENGINEERING IN MEDICINE AND BIOLOGY SOCIETY EMBC**	**1**	**1.010**
**2012 SIXTH INTERNATIONAL CONFERENCE ON SENSING TECHNOLOGY ICST**	**1**	**1.010**
**2014 IEEE HEALTHCARE INNOVATION CONFERENCE HIC**	**1**	**1.010**
**2015 IEEE INTERNATIONAL SYMPOSIUM ON MEDICAL MEASUREMENTS AND APPLICATIONS MEMEA PROCEEDINGS**	**1**	**1.010**
**2015 INTERNATIONAL CONFERENCE ON VIRTUAL REHABILITATION PROCEEDINGS ICVR**	**1**	**1.010**
**2017 39TH ANNUAL INTERNATIONAL CONFERENCE OF THE IEEE ENGINEERING IN MEDICINE AND BIOLOGY SOCIETY EMBC**	**1**	**1.010**
